# OPTCLOUD: An Optimal Cloud Service Selection Framework Using QoS Correlation Lens

**DOI:** 10.1155/2022/2019485

**Published:** 2022-05-25

**Authors:** Rakesh Ranjan Kumar, Abhinav Tomar, Mohammad Shameem, Md. Nasre Alam

**Affiliations:** ^1^Department of CSE, C V Raman Global University, Mahura, Jalna Bhubneshwar, Odisha, India; ^2^Department of CSE, Netaji Subhas University of Technology, New Delhi, India; ^3^Department of CSE, K L University, Guntur, Andhra Pradesh, India; ^4^Department of CSE, Woldia University, Woldia, Ethiopia

## Abstract

Cloud computing has grown as a computing paradigm in the last few years. Due to the explosive increase in the number of cloud services, QoS (quality of service) becomes an important factor in service filtering. Moreover, it becomes a nontrivial problem when comparing the functionality of cloud services with different performance metrics. Therefore, optimal cloud service selection is quite challenging and extremely important for users. In the existing approaches of cloud service selection, the user's preferences are offered by the user in a quantitative form. With fuzziness and subjectivity, it is a hurdle task for users to express clear preferences. Moreover, many QoS attributes are not independent but interrelated; therefore, the existing weighted summation method cannot accommodate correlations among QoS attributes and produces inaccurate results. To resolve this problem, we propose a cloud service framework that takes the user's preferences and chooses the optimal cloud service based on the user's QoS constraints. We propose a cloud service selection algorithm, based on principal component analysis (PCA) and the best-worst method (BWM), which eliminates the correlations between QoS and provides the best cloud services with the best QoS values for users. In the end, a numerical example is shown to validate the effectiveness and feasibility of the proposed methodology.

## 1. Introduction

With the advent of service computing, cloud computing has evolved into a growing computing paradigm that is revolutionizing the way of managing and delivering computing, storage, and on-demand service solution [1]. Cloud computing offered three different service models to its user either infrastructure as a service, platform as a service, and software as a service (IaaS, PaaS, and SaaS). Cloud computing is categorized into four types: private cloud, public cloud, hybrid cloud, and community cloud [2, 3]. The cornerstone of cloud computing is that users can access cloud services from anywhere, at any time on a subscription basis. Cloud computing provides the “pay-as-you-use” pricing model, where cloud users are charged only for the consumed resources. Many of the world's largest IT firms (including IBM, eBay, Microsoft, Google, and Amazon) have moved their existing business solutions to the cloud because of the advantages it offers [4].

A growing number of cloud service providers (CSPs) now provide their customers with a wide range of options for selecting the best cloud service for their individual functional needs. Many CSPs offer identical services, but at varied pricing and quality levels, and with a wide range of additional options and features. However, a supplier may be cheap for storage but expensive for computing. Because of the wide range of cloud service options available, it can be difficult for customers to determine which CSP is best suited to meet their specific needs. Incorrect selection of a cloud service provider (CSP) can lead to service failure, data security or integrity breaches, and noncompliance with cloud storage standards in the future [5].

Cloud service selection usually involves matching customer needs to the features of cloud services offered by various CSPs. The growing number of CSPs and their variable service offerings, pricing, and quality have made it difficult to compare them and choose the best one for the user's needs. To determine which cloud service provider (CSP) is the greatest fit for a cloud user's needs, a wide range of evaluation criteria for distinct cloud services from multiple CSPs must be considered. Recently, multiattribute decision-making (MCDM) has come up as one of the most efficient decision-making tools, showing its potential to solve real-world problems [6–10]. Thus, selecting the best CSP is a difficult MCDM problem in which various choices must be reviewed and ranked using a variety of criteria based on specific user preferences [11–13].

In recent year, numerous effort has been dedicated to the cloud service selection problem [11, 14, 15]. In literature, the existing methodology rarely takes into account the correlation of QoS criteria. Indeed, QoS criteria are often correlated with each other, i.e., strong positive correlation between availability and successability and a strong negative correlation between response time and throughput [16]. If a cloud service has a short response time, it may also have high throughput. Due to this reason, weighted summation of QoS data may cause repetitive computation over QoS attribute information. If the number of QoS attributes become larger, then the degree of repetitive computation will be higher and it costs larger calculation time. In this situation, it is difficult for the existing cloud service selection method to assess the QoS value of the cloud service accurately and efficiently [17].

In this scenario, we suggested a new framework called “optimal cloud (OPTCLOUD)” for the assessment and selection of the best cloud service based on QoS values. The primary objective of the OPTCLOUD model is to reduce the size of selection criteria without significant information loss and to keep the cloud service evaluation process expressive and easy. In view of these challenges, we have introduced an efficient and accurate evaluation method for cloud services based on PCA-BWM. Here, PCA is used to reduce the data dimension and eliminate the correlation among QoS criteria while BWM is used to determine the weight of each QoS criterion based on user preference [18, 19]. Note that basic PCA calculates the weight of the principal component based on the QoS information dataset, which is too objective. For this reason, we integrated the BWM method with PCA, for simplifying the cloud service selection process. In general, this contribution provides a faster and more effective method to minimize the limitation of the previous studies, i.e., subjectivity, high computational requirement, and multicollinearity. To the best of our knowledge, this is the first time that PCA and BWM have been directly applied to cloud service selection problems.

The significant contributions of this paper are listed as follows:

1. A novel “OPTCLOUD” framework is suggested for measuring the cloud service alternatives as per their offered QoS criteria value.
2. A technique is proposed that is both efficient and reliable for removing correlations between QoS criteria in complicated decision-making problems.
3. The experimental results demonstrate the feasibility of the proposed methods.

The remainder of this article is organized as follows.  Section 2 talks about the related work. A motivational example is discussed in Section 3.  Section 4 introduces some background knowledge that is admissible to our work. The proposed cloud service ranking scheme is discussed in Section 5.  Section 6 explains the proposed methodology for optimal cloud service selection. In Section 7, a numerical case study and sets of the experiment are included to depict the feasibility of the proposed methodology. In addition, the results and their validation are discussed. At last, Section 8 discusses the concluding remarks and future scope.

## 2. Related Works

This section compares and contrasts our work to previous efforts in order to demonstrate how our approach differs from those already in use for cloud service selection. In general, all proposed techniques select the cloud services based on customer preferences and QoS criteria. These cloud service decision-making methods are classified into two categories: MCDM-based cloud service selection method and non-MCDM-based cloud service selection method.

### 2.1. MCDM-Based Cloud Service Selection Methods

A thorough review of the literature reveals that the application of MCDM-based techniques for cloud service selection and ranking has received a significant amount of attention. Some of the frequently used MCDM techniques such as analytic hierarchy process (AHP) [20], analytic network process [21], Technique for Order of Preference by Similarity to Ideal Solution (TOPSIS) [22], Simple Additive Weighting (SAW) [23], multiattribute utility theory [24], best-worst method (BWM) [19], and outranking [25]. Using a modified DEA and SDEA model, the author [26] shows how to pick the best cloud service from among a variety of options based on the needs of the customer. Using a fuzzy ontology, a new fuzzy decision-making framework is proposed in the paper [27], which is capable of handling fuzzy information and finding the best cloud service. The authors use fuzzy AHP and TOPSIS to calculate QoS weights and measure cloud service performance. Lang et al. [28] proposed a Delphi method for identifying and classifying the QoS criteria for cloud service provider evaluation. In [29], a framework called TRUSS was presented for the identification of trustworthy cloud services. A brokerage-based cloud service selection framework was proposed in the paper [30]. Ding et al. [31] came up with collaborative filtering to make service recommendations that are time-sensitive. The objective was to expedite the process of identifying cloud service providers with a higher level of customer satisfaction. These decision-making methods can be divided into stochastic, deterministic, and fuzzy methods depending on the type of data they use and how many decision-makers participate in the decision process, i.e., single or multiple (group).  Table 1 presents the overview of the cloud service selection-related work based on the MCDM method.

### 2.2. Non-MCDM-Based Cloud Service Selection Methods

In this subsection, we review the existing literature on service selection methods that are not based on MCDM. These methods include optimization techniques, game theory, graph theory, descriptive logic, collaborative filtering, and linear programming.

Lang et al. [28] proposed a Delphi method for identifying and classifying the QoS criteria for cloud service provider evaluation. In [29], a framework called TRUSS was presented for the identification of trustworthy cloud services. A brokerage-based cloud service selection framework was proposed in the paper [42]. Somu et al. [43] suggested a method for cloud services based on hypergraphs. Somu et al. [44] developed a preliminary set-based hypergraph technique for service selection. In the paper [45], the author suggested a method for identifying the best cloud service by excluding less dependable services based on QoS criteria. Ding et al. [31] came up with collaborative filtering to make service recommendations that are time-sensitive. The objective was to expedite the process of identifying cloud service providers with a higher level of customer satisfaction. For nonfunctional requirements, Ma et al. [39] suggested a collaborative QoS model for identifying the optimal cloud service. Wang et al. [46] developed a game-theoretic strategy for QoS-aware cloud service selection.

### 2.3. Differences between Our Research and Existing Work

To the best of our knowledge, there are a few research studies that have a correlation between QoS attributes. In the paper [47], the author considers the correlation between different QoS attributes but how to handle these correlations is not discussed. A business service-based correlation model is discussed in the paper [48]; however, it only discusses modeling aspects and the model is not applied in the service selection process. A PCA-based Web service selection method is discussed in the paper [16]. By using the PCA method, this paper tries to eliminate the correlation between QoS attributes but the author does not consider the QoS attribute weight information that plays an important role in the service selection process. The paper [17] discusses a method for selecting multimedia services based on weighted PCA. However, two shortcomings exist in this paper. First, this paper still lacks adaptability in weight assignment for QoS attributes, i.e., how to assign appropriate QoS weight information accurately and efficiently. Second, this paper used uniform standardization for all QoS attributes that bring discrepancies between negative and positive attributes.

To address the shortcoming of existing work, we have developed a PCA-BWM-based scheme for optimal cloud service selection based on correlation aspects. Our work differs in several ways from the existing cloud service selection methodology. On the one hand, we use the PCA method to exclude the correlation between different service quality attributes and reduce the impact of false or artificial QoS attribute information. On the other hand, in order to assign a weight to the various QoS attributes efficiently and precisely, we use the BWM method. The BWM is a subjective method that considers the subjective preference of cloud customers in the cloud service selection process. This study combines the objective and subjective aspects to achieve a better assessment result in cloud service selection problems.

## 3. Motivational Example

Suppose *n* cloud service providers satisfy the cloud customer's functional requirement and are ready to deliver their services with QoS parameters, i.e., availability, latency, response time, throughput, etc. These QoS parameters are not independent but correlate with one another. In this situation, selecting an efficient and accurate cloud service among *n* service providers based on these QoS criteria becomes a challenging task for a cloud user.

**Figure 1:**
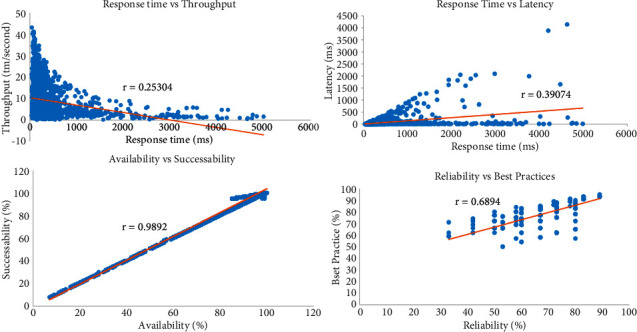
Correlation between QoS attributes for QWS dataset.

Our hypothesis was tested using a real-world QWS dataset of 2507 real services with 9 QoS parameters, including response time (RT), availability (Ava), successability (Succ), throughput (Th), reliability (Re), compliance (Com), best practices (BP), latency (Lat), and documentation (Doc) [49]. We computed the correlation coefficient matrix of the QWS dataset as displayed in Table 2. We define the correlation coefficient for the QoS parameters as a regression line and this is displayed in Figure 1. In this figure, we can observe a strong positive correlation between successability and availability at a value of 0.9892. Moreover, there is a positive correlation between best practice and reliability at a value of 0.6895. Furthermore, the correlation between response time and throughput shows the strongest negative correlation at a value of −0.2530. This finding indicates that certain QoS parameters associated with cloud services are not independent but rather correlated. As a result, the current cloud service selection methods are ineffective in this case, leading to an inaccurate result when selecting cloud services.

## 4. Background Knowledge

### 4.1. Best-Worst Method

Razaei introduced the best-worst method (BWM) in 2015 as a new MCDM method for determining the relative importance of QoS criterion weights [19]. Compared to other well-known MCDM methods like AHP, this novel method finds more consistent results with fewer pairwise comparisons [20]. In the AHP method, if there are a number of QoS criteria, then *n*(*n* − 1)/2 comparisons are required while, in the BWM, only 2*n* − 3 comparisons are needed.

### 4.2. Principal Component Analysis

PCA is a powerful multivariate statistical procedure proposed by Pearson [18]. It is a dimensionality reduction technique which decreases the dimension of a dataset without much loss of information [50]. PCA transforms a large number of interrelated variables into a set of values of linearly uncorrelated variables, which are known as principal components. The principal components are calculated by identifying the eigenvalue of a covariance matrix of the original dataset. Note that the number of principal components can be equivalent to or less than the original variables. This conversion is performed so that the first main component maintains maximum variance and all succeeding components have remaining variance without being correlated with the preceding components.

## 5. The Proposed Cloud Service Ranking Scheme

The objective of this study is to introduce a new cloud service selection scheme among the available cloud service alternatives according to the needs of cloud users. The proposed scheme has three major parts: (a) the basic idea of the cloud service selection scheme, (b) the OPTCLOUD framework, (c) and the schematic diagram. This section focuses on the detailed description of the proposed framework and explores the schematic diagram. The following subsection describes the detailed explanation of each part.

### 5.1. Basic Idea

In essence, a cloud service selection problem leverages the correlation information among various QoS attributes which involves objective and subjective aspects. Based on the QoS value of cloud service, objective aspects and subjective aspects are collected based on cloud customer preference over different QoS attributes.

To assess the objective aspect of the QoS attribute value, the PCA method is used to minimize dimensionality through the analysis of covariance between the QoS attributes. It transforms a series of correlated QoS attributes into an independent principal component without losing too much information.

Apparently, the only objective aspect is not enough to find a suitable cloud service. Because individual preference over different QoS attributes plays an important role in cloud service selection problems, we calculate the weight of the QoS attributes in our proposed scheme by applying the best-worst method (BWM). It has more consistent results and has less pairwise comparison than the popular AHP method. Finally, the integration of BWM and PCA methods provides a trade-off between computational complexity and results in a more rational way with consistency in the selection of cloud services.

### 5.2. Proposed OPTCLOUD Framework

**Figure 2:**
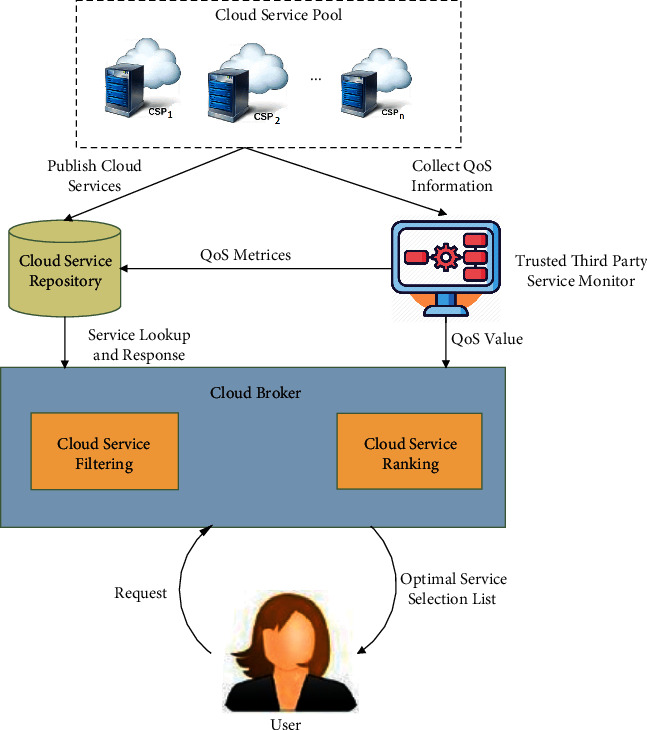
OPTCLOUD framework for cloud service selection.

This section introduces a proposed broker-based framework (OPTCLOUD) for cloud service as shown in Figure 2. This framework consists of three distinct components: (i) cloud broker, (ii) cloud benchmark service provider, and (iii) cloud service repository. The framework is reliant on the quality of service (QoS) information that comes from multiple sources. The service provider's specifications and third-party monitoring services are examples of these sources. Furthermore, we also suppose that the cloud service repository keeps track of available cloud service providers. The thirst party monitoring services keep track of all of the cloud services that have been registered. In order to collect QoS performance data, the thirst party monitoring services run benchmark tests against all of the cloud services that are available. The performance data are kept in the QoS repository, and QoS performance data is used by cloud brokers to recommend appropriate cloud services to users.

- Cloud broker: the suggested framework is built around the cloud broker. It performs a variety of activities, as illustrated in Figure 2, including cloud service discovery and cloud service ranking. It interacts with the cloud service repository to filter the cloud services that meet the cloud user's requirements. The cloud broker's cloud service ranking module ranks filtered cloud services according to the significance of each QoS parameter provided by the cloud user. For each cloud service, a ranking is generated using the proposed methodology. The cloud service discovery module is used to discover and store information about the various cloud services available.
- Cloud benchmark service provider: this component is a third party that audits or monitors cloud services continuously. It performs benchmark tests on available cloud services on QoS criteria such as availability, reliability, throughput, and efficiency. It goes through an extensive testing process on a QoS criterion several times in order to verify the QoS claims made by the cloud service providers. Cloud benchmark service providers such as CloudSpectator [10], CloudHarmony [111], and others analyze cloud services on a regular basis and put the results in a cloud service repository.
- Cloud service repository: it is a database that store information about cloud service providers and their services on various QoS attributes. This is where data from cloud providers and third-party monitoring services on service standards and performance is stored. This repository is used for prescreening services by cloud broker when they are looking for candidate services that meet their customer's needs.

### 5.3. Schematic Diagram

**Figure 3:**
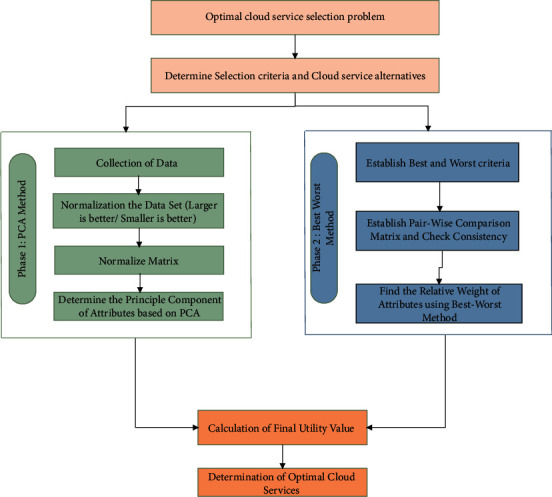
Methodology of cloud service selection.

Figure 3 shows the suggested schematic diagram. The overall process of cloud service selection includes four important steps:

1. Determine the evaluation criteria and cloud service alternatives used in the service selection process.
2. Utilize the best-worst method to calculate the weight of the evaluation criteria.
3. Eliminate correlation between QoS criteria using the PCA method.
4. Combine BWM and PCA to evaluate the cloud service alternatives and rank them based on performance values.

## 6. The Proposed Cloud Service Selection and Ranking Methodology

We have illustrated the suggested methodology in this section, using a schematic diagram followed by a detailed procedure to select the appropriate cloud services alternative among the eligible alternatives.

### 6.1. Cloud Service Selection Methodology

In this subsection, we introduced PCA-BWM-based techniques for selecting the best cloud service among many available cloud alternatives.

#### 6.1.1. Construct a Decision Matrix

We create a decision matrix DM of *m* ∗ *n*, in which *m* represents the eligible cloud service alternatives denoted by (CSP*i*) that satisfy the cloud customer's functional and nonfunctional requirements, and *n* represents the number of QoS criteria for determining the best cloud service provider. It is shown in

(1) $$\begin{array}{c} \text{DM}=\left[\begin{array}{ccc} x_{11} & \ldots & x_{1n}\\ \vdots & \ddots & \vdots \\ x_{m1} & \ldots & x_{mn} \end{array}\right], \end{array}$$

where *x*_*ij*_ represents the QoS value delivered by (CSP_*i*_) on QoS criteria *j*.

#### 6.1.2. Apply Best-Worst Method for QoS Criteria Weight Calculation

In the decision-making process, the calculation of the weights of criteria is a critical phase that has a direct impact on the ranking of alternatives. In most cases, the criteria are not given equal weight. Each criterion has a different value depending on the needs of a cloud user. Because of this, determining the weight of each criterion is essential. The majority of authors, according to the literature, employ the AHP approach to calculate the weights of the criterion. However, due to AHP's limitations, we recommend that the weights of the criterion be computed using BWM.

According to Rezaei [6], MCDM problems need to be evaluated against a set of criteria in order to choose the best option. However, BWM works in a different way. It is a process in which the decision-maker identifies the best and worst criteria. Decision-makers make the decision by comparing the best/worst criteria and other criteria in pairwise comparison. In the BWM method, a linear programming max-min problem is used to determine criterion weights. Using the CR, the decision-maker can confirm the validity of the criterion weight.

The best-worst technique is used to determine the weight of QoS criteria. It consists of six steps:

**Step 1 .**

Make a list of criteria. The decision-maker establishes a list of *n* criteria against which all potential cloud service providers will be evaluated.

**Step 2 .**

The cloud customer and the decision-maker work together to figure out which QoS criteria are the best (or the most preferred) and which are the worst (or the least preferred) out of all of the other criteria.

**Step 3 .**

To estimate the best criterion's preference over all other criteria (best-to-others), use a scale of 1–9 as given in Table 3. The preference vector as a result would look like

(2) $$\begin{array}{c} A_{B}=\left(a_{B1},a_{B2},\ldots ,a_{Bn}\right), \end{array}$$

where *a*_*Bj*_ depicts the priority of the best criterion *B* over all other criteria *j* and *a*_BB_=1.

**Step 4 .**

Similar to the above, using Table 3, determine the priority of all other decision criteria against the worst criteria. The resulting preference vector would be

(3) $$\begin{array}{c} A_{W}={\left(a_{1W},a_{2W},\ldots ,a_{nW}\right)}^{T}, \end{array}$$

where *a*_*jW*_ denotes the preference of *jth* criterion over the worst criterion *W* and *a*_WW_=1.

**Step 5 .**

Finally, by solving (4) and (5), the optimal weights for each criterion are determined, which is represented by the vector (*w*_1_^*∗*^, *w*_2_^*∗*^,…, *w*_*n*_^*∗*^) associated with each QoS criterion (*c*_1_, *c*_2_,…, *c*_*n*_).

(4) $$\begin{array}{c} \left|\frac{w_{B}}{w_{j}}-a_{Bj}\right|\leq \varepsilon \;\text{for all }j, \end{array}$$

(5) $$\begin{array}{c} \left|\frac{w_{j}}{w_{w}}-a_{jW}\right|\leq \varepsilon \;\text{for all }j, \end{array}$$

(6) $$\begin{array}{c} \sum_{j}w_{j}=1\;w_{j}>0,\;\text{for all }j. \end{array}$$

Here, *ε* is the optimum value required to estimate the consistency ratio of pairwise comparisons. The weight of the best QoS criterion is *w*_*B*_, and the weight of the worst criterion is *w*_*W*_.

**Step 6 .**

Use (7), to calculate the consistency ratio (CR).

(7) $$\begin{array}{c} \text{CR}=\frac{\varepsilon }{\text{CI}}, \end{array}$$

where CI denotes the consistency index, which is depicted in Table 4. The consistency ratio ranges from 0 to 1. CR values near zero are considered more consistent, while those near one are considered less so.

#### 6.1.3. PCA-BWM-Based Cloud Service Selection Method

The various steps of the proposed PCA-BWM methodology are described as follows.

**Step 7 .**

Standardize the original decision matrix.

Due to their vast diversity, the values of QoS criteria are estimated in different measuring units and ranges. This may lead to inconsistency during comparison. Therefore, in this step, each QoS criterion value of the matrix DM is normalized to accomplish a uniform comparison.

We have classified the QoS criteria into positive and negative criteria. The positive criteria include throughput, availability, and reputation, whereas the negative criteria include response time and cost. Note that a higher value of the QoS criterion signifies the higher quality of positive criteria and the low quality of negative criteria. Thus, we must normalize them separately in order to eliminate the inconsistency between negative and positive criteria. For this purpose, we have used the max-min normalization approach. This normalization approach converts each criteria value to the same scale.

The positive and negative criteria are normalized using (8) and (9):

(8) $$\begin{array}{c} nx_{i,j}=\left\{\begin{array}{c} \frac{x_{ij}-x_{j}^{\min}}{x_{j}^{\max}-x_{j}^{\min}},\;\text{if }x_{j}^{\max}\neq x_{j}^{\min},\\ 1\;\text{if }x_{j}^{\max}=x_{j}^{\min}, \end{array}\right. \end{array}$$

(9) $$\begin{array}{c} nx_{i,j}=\left\{\begin{array}{c} \frac{x_{j}^{\max}-x_{ij}}{x_{j}^{\max}-x_{j}^{\min}},\;\text{if }x_{j}^{\max}\neq x_{j}^{\min},\\ 1\;\text{if }x_{j}^{\max}=x_{j}^{\min}, \end{array}\right. \end{array}$$

where *x*_*j*_^min^ and *x*_*j*_^max^ are the minimum and maximum values of the *j*^*th*^ QoS criterion among all the cloud services. The normalized decision matrix *N* is represented as

(10) $$\begin{array}{c} N=\left[\begin{array}{cccc} nx_{11} & nx_{12} & \ldots & nx_{1n}\\ nx_{21} & nx_{22} & \ldots & nx_{2n}\\ \vdots & \vdots & \vdots & \vdots \\ nx_{n1} & nx_{n2} & \ldots & nx_{nn} \end{array}\right]. \end{array}$$

**Step 8 .**

Calculate the correlation coefficient matrix.

In this step, we have computed the correlation coefficient matrix *R*=(*r*_*ij*_)_*n∗n*_ of matrix *N*, where *r*_*ij*_ reflects the correlation coefficient between *nx*_*i*_ and *nx*_*j*_ and which can be denoted as

(11) $$\begin{array}{c} r_{ij}=\frac{\sum_{k=1}^{n}\left(nx_{kj}-nx_{i}\right)\left(nx_{kj}-nx_{j}\right)}{\sqrt{\sum_{k=1}^{n}{\left(nx_{kj}-nx_{i}\right)}^{2}\sum_{k=1}^{n}{\left(nx_{kj}-nx_{j}\right)}^{2}}}. \end{array}$$

**Step 9 .**

Calculate the eigenvalues and eigenvector of the correlation coefficient matrix and find the principal component.

By using the characteristic equation |*λI* − *R*|, we calculated *p* eigenvalues (*λ*) of the correlation matrix *R* and sort them as *λ*_1_ ≥ *λ*_2_ ≥ …≥*λ*_*p*_ ≥ 0. We also calculated the eigenvector *u*_*i*_ corresponding to the eigenvalues *λ*_*i*_, *i*=1,2,…, *p*, and normalized it so that ∑_*j*=1_^*p*^*u*_*ij*_^2^=1 where *u*_*ij*_ is the *j*^*th*^ component of eigenvector *u*_*i*_.

**Step 10 .**

Compute the contribution ratio and cumulative contribution ratio of the principal components.

In this step, we have calculated the contribution ratio and the cumulative contribution ratio using (12) and (13), respectively.

(12) $$\begin{array}{c} T_{k}=\frac{\lambda_{k}}{\sum_{i=1}^{p}\lambda_{i}}, \end{array}$$

(13) $$\begin{array}{c} E=\frac{\sum_{k=1}^{m}\lambda_{i}}{\sum_{i=1}^{p}\lambda_{i}}. \end{array}$$

We selected first *m* principal components *λ*_1_, *λ*_2_,…, *λ*_*m*_(*m* ≤ *p*), whose cumulative contribution value is higher than 85%. We found the principal component *z*_*k*_=∑_*j*=1_^*p*^*u*_*kj*_*∗N*, where *k*=1,2,…, *p* and *N* is a normalized matrix. Final principal components are denoted as *z*=(*z*_1_, *z*_2_,…, *z*_*m*_) and would be used to replace the original QoS criteria (*Q*_1_,…, *Q*_*p*_). These principal components are independent of each other and simplify the cloud service selection process with accurate results. We computed the comprehensive evaluation value of the principal component using

(14) $$\begin{array}{c} z_{k}=\sum_{i=1}^{m}\left[\frac{\lambda_{k}}{\sum_{i=1}^{p}\lambda_{i}}\right]*\sum_{j=1}^{p}u_{kj}N. \end{array}$$

**Step 11 .**

Integrate the BWM weight of criteria with PCA and determine the final comprehensive value.

We have calculated a weighted normalized matrix *N*_*j*_^*∗*^=*w*_*j*_*N*_*j*_, where *j* = 1,2,…, p and *w*_*j*_ is the weight of jth QoS criteria determined using the BWM method. So, the new principal component is ∑_*j*=1_^*p*^*u*_*kj*_*N*^*∗*^. Finally, we calculated the total comprehensive value determined by PCA using

(15) $$\begin{array}{c} Z_{k}=\sum_{i=1}^{m}\left[\frac{\lambda_{k}}{\sum_{i=1}^{p}\lambda_{i}}\right]*\sum_{j=1}^{p}u_{kj}N^{*}. \end{array}$$

Here, we have sorted the final values in ascending order and ranked them based on comprehensive values. The cloud service having the highest comprehensive value will be selected as the best cloud service.

## 7. Case Study with Experiments Analysis

This section evaluates the proposed methodology's efficacy using a real-world QoS dataset. Cloud services share many aspects with Web services, particularly in terms of quality of service (QoS); hence, we have utilized the publicly available QWS dataset as a benchmark for cloud service. This dataset was created by Eyhab Al-Masri of Guelph University [49]. The QWS dataset has been widely accepted across the research community and used in evaluation studies based on the QoS service selection problem. The QWS dataset includes 2507 real Web services with their quality values over nine parameters such as throughput, availability, response time, reliability, latency, scalability, best practices, compliance, and documentation. The QWS dataset is comprised of a variety of Web services that were collected from the real Web using the Web Service Crawler Engine (WSCE). All of these Web services were obtained from publicly available sources on the Internet, such as service portals, search engines, and UDDI registries. In our experiments, we only use version 1.0 of the QWS dataset because it provides ratings and classification. Version 1.0 of the QWS dataset contains 364 Web services, each of which has a set of nine quality factors tested using commercial benchmark tools. Using the QWS dataset, the following subsection illustrates a case study. Following this, an experiment is carried out to test the practicality of the proposed methodology. The results of the experiment demonstrate that our methodology outperforms all other methods.

### 7.1. An Illustrative Case Study

#### 7.1.1. Introduction

For this case study, we looked at 10 alternative cloud services from the QWS dataset that had the same functionality. Eight quality criteria are used to evaluate these services, i.e., the response time (Q1), availability (Q2), throughput (Q3), successability (Q4), reliability (Q5), compliance (Q6), best practices (Q7), and latency (Q8). The response time and the latency of these quality criteria are considered negative criteria and the remaining are treated as positive criteria. We refer to ten cloud services by the acronyms CSP1, CSP2,…, and CSP10. This makes it easier to talk about them.  Table 5 shows a decision matrix with ten cloud service alternatives and eight QoS criteria.

#### 7.1.2. Find Relative Weight of QoS Attributes Using BWM Method

At this point, we have utilized the BWM to determine the relative importance of eight quality of service criteria. From all QoS criteria, the best and the worst are determined with the help of the cloud user. Here, we assume that the best criterion is response time (*Q*1) and that the worst criterion is throughput (*Q*3). Relative preference (between 1 and 9) is given for the best criteria over all other criteria (*Q*1, *Q*2, *Q*3, *Q*4, *Q*5, *Q*6, *Q*7, *Q*8) and also provides a relative preference of other criteria over the worst criteria, as shown in Table 6. We obtained the weights of the criteria using (4) and (5). The weights obtained are *Q*1 = 0.276, *Q*2 = 0.174, *Q*3 = 0.028, *Q*4 = 0.116, *Q*5 = 0.063, *Q*6 = 0.109, *Q*7 = 0.152 and *Q*8 = 0.082, and *ε*  = 0.042. Equation (7) is used to find out the consistency ratio, and the value is CR = 0.01, which indicates high consistency.

#### 7.1.3. Application of PCA-BWM Method

Here, each QoS criterion is different in terms of unit and range. To remove inconsistencies in QoS information of cloud services, the normalization is performed by using the max-min normalization, and the positive and negative criteria are standardized separately with (8) and (9). The normalized matrix of these cloud services is shown in Table 7. The calculated correlation coefficient matrix between QoS criteria using (11) is shown in Table 8. This table shows a strong positive correlation (0.9769) between successability and availability while a negative linear correlation (−0.488) between response time and throughput.

**Figure 4:**
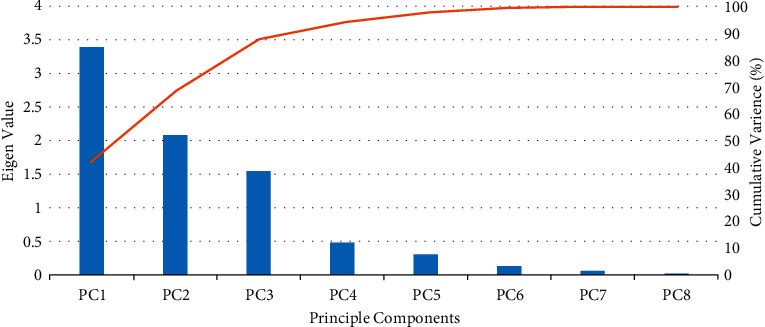
PCA analysis for Cloud service selection.

Table 9 illustrates the eigenvalue of the correlation matrix, its contribution ratio, and cumulative contribution ratio. Here, we can see that the cumulative contribution rate of the first three components reaches up to 87.22% (Figure 4), which is high enough. Therefore, the first three principal components *z*_1_, *z*_2_, *z*_3_ replace the other criteria to do a comprehensive evaluation as shown in Table 10. Thus, we successfully reduce the evaluation criteria from 8 to 3 which are independent of each other.

Now, the values of the three independent principal components *z*_1_, *z*_2_, and *z*_3_ are calculated as follows:

(16) $$\begin{array}{c} z_{1}=-0.26N_{1}^{*}+0.481N_{2}^{*}-0.1034N_{3}^{*}+0.4725N_{4}^{*}-0.4101N_{5}^{*}+0.3217N_{6}^{*}-0.3358N_{7}^{*}+0.2875N_{8}^{*},\\ z_{2}=0.1475N_{1}^{*}-0.2865N_{2}^{*}-0.3829N_{3}^{*}-0.2914N_{4}^{*}-0.3088N_{5}^{*}-0.4065N_{6}^{*}-0.4231N_{7}^{*}-0.4744N_{8}^{*},\\ z_{3}=0.6426N_{1}^{*}+0.0331N_{2}^{*}+0.5942N_{3}^{*}-0.0405N_{4}^{*}-0.2756N_{5}^{*}+0.1751N_{6}^{*}-0.1033N_{7}^{*}-0.3376N_{8}^{*}. \end{array}$$

We find a new evaluation function, i.e., FUNC_*Z*(*z*_1_, *z*_2_, *z*_3_)  =  0.4228*z*_1_+0.2605*z*_2_+0.1933*z*_3_. Now, we calculate the comprehensive value (*Z*) using the evaluation function and the results are shown in Table 11. The cloud service alternatives have a higher score of the comprehensive value selected as the best cloud service alternatives.

### 7.2. Experiments

In this subsection, we carried out a set of experiments to evaluate the suitability of the proposed methodology. For these experiments, we generated some artificial data with QWS data set, by varying the cloud service provider and QoS criteria.

#### 7.2.1. Comparison with Other Existing MCDM Methods

**Figure 5:**
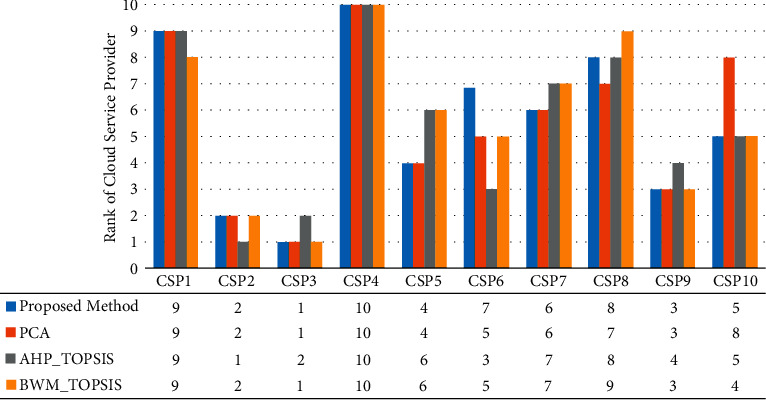
Ranking of cloud service providers with different methods.

We compared our results with other popular MCDM methods, namely, PCA, BWM_TOPSIS, and AHP_TOPSIS [17, 33, 51].  Figure 5 shows the experimental result of different methods. In most cases, there is a clear resemblance between the outcomes acquired through the suggested methodology and other techniques. Therefore, the results of the proposed approach can be concluded as accurate and precise.

#### 7.2.2. Measuring Execution Time with respect to the Number of QoS Attributes

**Figure 6:**
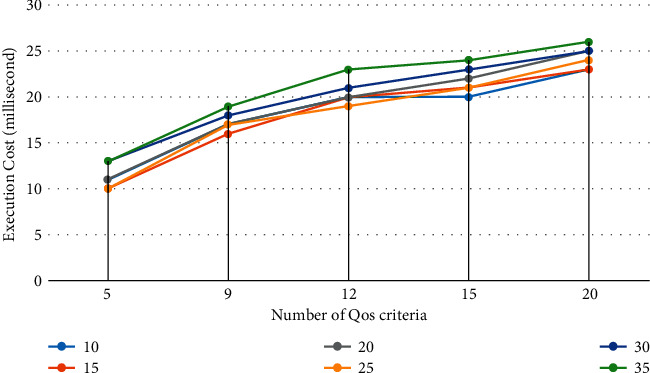
Execution cost with respect to no QoS attributes.

This experiment evaluates the average execution time of the proposed methodology based on different QoS criteria. The experimental results are shown in Figure 6. In this experiment, the number of QoS criteria varies between 3 and 20, and the number of cloud service providers varies between 10 and 35. We can see that the execution time increases slowly with the increase in the number of cloud service providers, which indicates that the running time costs are not more affected by the number of cloud service providers. From Figure 6, the running time costs are more affected by increasing the number of QoS criteria. This is because the covariance matrix and the priority weight are obtained by considering all QoS criteria.

#### 7.2.3. Reduce Dimensionality of the Selection Criteria

**Figure 7:**
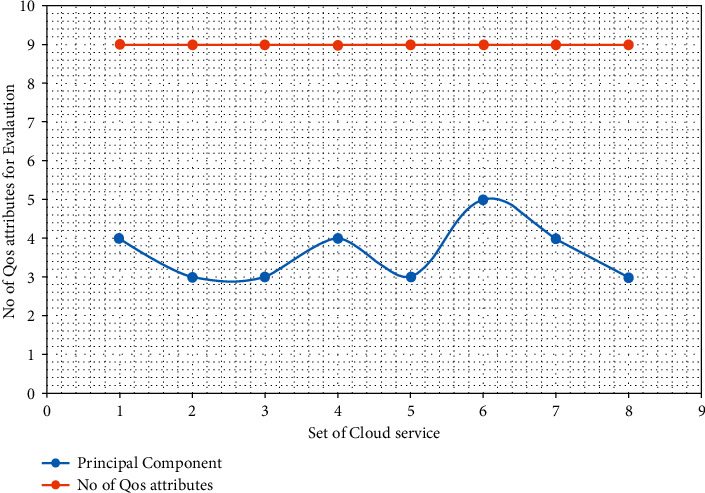
The number of principal components versus the number of QoS attributes.

We know that a large number of QoS criteria are involved in the cloud service selection process that is difficult to handle without an advanced computer program. The primary goal of the proposed OPTCLOUD framework is to reduce the dimensionality of selection criteria without significant information loss and to keep the cloud service evaluation process simple. In this experiment, we use the data of eight cloud services from QWS data sets. The experimental results are shown in Figure 7. Here, we can see that the principal components are always smaller than the number of original QoS criteria. These results confirm that the proposed methodology reduces the evaluation criteria and simplifies the cloud service selection process.

#### 7.2.4. Sensitivity Analysis of Result

This subsection validates the robustness and efficiency of the suggested scheme using sensitivity analysis. To carry out the sensitivity analysis, we check how the cloud service provider's ranking may change under different weight values. In this scenario, we execute the whole process to monitor the changes in various circumstances. The ranks of cloud service providers are determined for each case by evaluating the effect of changes in criterion weight.

**Figure 8:**
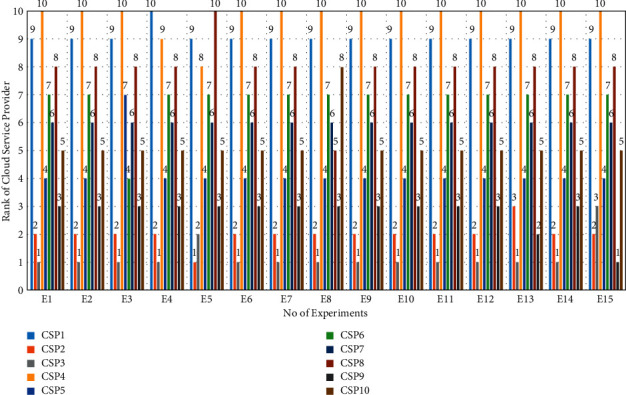
Result of sensitivity analysis.

We conducted a sensitivity analysis by swapping the weights of each of the nine criteria for the weights of another criterion. Therefore, we created fifteen distinct experiments. We assigned a unique name to each experiment (E1 ldots E15). During each experiment, we used data from our case study to run the proposed methodology and collect data about how it worked (Section 7).  Figure 8 shows the outcomes of 15 experiments. CSP3 emerged as the best service in 14 out of 15 experiments, as shown in Figure 8. For second place, CSP2 was preferable to the other in 13 out of 15 studies. Finally, sensitivity analysis shows that the rank of cloud service providers is proportional to the weight of the associated criteria. Therefore, we can infer that the suggested method is reliable and rationally ranks alternatives in accordance with preferences expressed by stakeholders.

## 8. Conclusion

Finding the best cloud service for cloud users is a challenge if there are many QoS criteria. In general, most of the QoS criteria are correlated and are ignored by the existing works. In this study, we analyzed the effects of the correlation of QoS criteria and proposed a novel cloud service selection methodology that combines PCA and BWM. The proposed work differs in many ways from the existing research works. First, we reduced the number of QoS criteria to simplify the process of selecting cloud services. Secondly, it removes the correlation between different QoS criteria and produces more authentic selection results. This contribution provides a new OPTCLOUD framework for the cloud service selection process. The proposed scheme demonstrates its feasibility and efficiency through a series of experiments with real datasets. Finally, we make a comparison with the other method to show that the proposed methodology outperforms them. However, the proposed work has some shortcomings. The proposed methodology only retains 87.22% of the total information, which represents a significant loss of information. This opens a possible future extension of our work. Our future efforts will be to improve PCA and simultaneously reduce dimensionality by losing the minimum amount of information.

## Figures and Tables

**Table 1:** MCDM-based cloud service selection approaches.

Reference	Selection and ranking approach	Brief description	Validation
Garg et al. [11]	AHP based ranking	Developed a framework called SMICloud to rank cloud services based on functional and nonfunctional QoS parameters	CS
Tripathi et al. [32]	ANP	Proposed SMI framework based QoS criteria interactions for the ranking of the cloud services	CS
Singh and sidhu [33]	AHP and improved TOPSIS	Proposed a framework to evaluate the trustworthiness of cloud service providers based on various QoS criteria	SA
Jaiswal and mishra [34]	Fuzzy ontology and MCDM method	Proposed a framework that models nonlinear preferences of users based on criteria interactions for cloud service selection and ranking	EA
Kumar et al. [12]	AHP and TOPSIS	Designed a new framework to rank cloud services in a crisp environment	CS/SA
Nawaz et al. [35]	Markov chains and BWM	Proposed brokerage-based architecture for the selection of cloud services based on user priorities	EV
Basset et al. [36]	Neutrosophic set theory with AHP	A proposed new multicriteria decision-making model to select suitable cloud service provider	CS
Yadav and goraya [37]	AHP	Developed a framework to handle QoS requirements of cloud customer	CS
Jatoth et al. [38]	AHP and grey TOPSIS	Apply AHP to compute the importance of QoS parameters and integrated grey set theory with TOPSIS to rank the cloud services	CS
Ma et al. [39]	Collaborative filtering with TOPSIS	Proposed a method which considers the objective QoS variation and subjective user preferences during different time periods	CS
Sun et al. [27]	Fuzzy ontology and MCDM method	Developed a framework that models nonlinear preferences of users based on criteria interactions for CSRS	EA
Hussain et al. [40]	Best-worst method	Perform services evaluation from a QoS perspective and overcome the drawbacks of AHP	EA
Hussain et al. [40]	Fuzzy linear best-worst method	Proposed FLBWM method which recommends appropriate cloud service to clients based on their QoS requirements	CS
Tiwari et al. [13]	Neutrosophic set theory and TOPSIS	Proposed a framework that integrated neutrosophic set theory with modified TOPSIS for ranking cloud services	CS
Kumar et al. [41]	Fuzzy AHP and TOPSIS	Proposed a fuzzy framework for the selection of cloud services	EA/SA

EA: experimental analysis, CS: case study, SA: sensitivity analysis.

**Table 2:** Correlation coefficient of QWS dataset QoS attributes.

	RT	Ava	Succ	Th	Re	Com	BP	Lat	Doc
RT	1								
Ava	−0.0664	1							
Succ	−0.2530	0.2007	1						
Th	−0.0773	0.9892	0.2007	1					
Re	0.0471	0.1289	0.2556	0.1211	1				
Com	−0.0828	0.2436	0.0603	0.2609	−0.03	1			
BP	0.0327	0.0571	0.1684	0.0554	0.6895	0.0336	1		
Lat	0.3907	−0.0988	−0.1450	−0.1107	−0.0239	−0.0773	−0.0079	1	
Doc	−0.0402	−0.0058	−0.0311	0.0044	0.0606	−0.0803	−0.0366	−0.0403	1

**Table 3:** Assessment scale.

Value	1	3	5	7	9	2,4,6,8
Description	Equal priority	Moderate priority	Strong priority	Very strong priority	Absolute priority	Intermediate values

**Table 4:** Consistency index (CI) value.

*a*_BW_	1	2	3	4	5	6	7	8	9
CI	0	0.44	1.00	1.63	2.3	3	3.73	4.47	5.23

**Table 5:** Decision matrix for ten cloud services.

Cloud service	*Q*1	*Q*2	*Q*3	*Q*4	*Q*5	*Q*6	*Q*7	*Q*8
CSP1	307.75	71	2.1	71	73	78	84	2.37
CSP2	498.5	91	4.8	91	60	89	82	31.17
CSP3	283.74	86	3.3	87	53	89	66	96.78
CSP4	130.33	63	7.8	63	73	78	84	14.66
CSP5	3610.2	96	1.4	99	67	100	77	6.4
CSP6	1314.75	78	3.5	79	73	78	84	30.75
CSP7	2561.33	87	1.2	96	67	78	72	28
CSP8	297.38	71	1.9	72	73	89	75	6.38
CSP9	498.5	91	4.8	91	60	89	82	31.17
CSP10	305.4	93	12.2	98	73	100	84	7.8

**Table 6:** Pairwise comparison for best-to-others (BO) and others-to-worst (OW).

Best-to-other (BO)	*Q*1	*Q*2	*Q*3	*Q*4	*Q*5	*Q*6	*Q*7	*Q*8
Best criteria: response time (*Q*1)	1	2	8	3	5	3	2	4
Others-to-worst (OW)	Worst criteria: throughput (*Q*3)							

*Q*1	8							
*Q*2	5							
*Q*3	1							
*Q*4	2							
*Q*5	4							
*Q*6	5							
*Q*7	6							
*Q*8	2							

**Table 7:** Normalized decision matrix.

Cloud service	*Q*1	*Q*2	*Q*3	*Q*4	*Q*5	*Q*6	*Q*7	*Q*8
CSP1	0.1898	0.0485	0.0049	0.0444	0.03	0	0.04	0.04
CSP2	0.1788	0.1697	0.0196	0.1556	0.0105	0.015	0.0356	0.0278
CSP3	0.1912	0.1394	0.0115	0.1333	0	0.015	0	0
CSP4	0.2	0	0.036	0	0.03	0	0.04	0.0348
CSP5	0	0.2	0.0011	0.2	0.021	0.03	0.0244	0.0383
CSP6	0.1319	0.0909	0.0125	0.0889	0.03	0	0.04	0.028
CSP7	0.0603	0.1455	0	0.1833	0.021	0	0.0133	0.0291
CSP8	0.1904	0.0485	0.0038	0.05	0.03	0.015	0.02	0.0383
CSP9	0.1788	0.1697	0.0196	0.1556	0.0105	0.015	0.0356	0.0278
CSP10	0.1899	0.1818	0.06	0.1944	0.03	0.03	0.04	0.0377

**Table 8:** Correlation coefficient between QoS attributes.

Cloud service provider	*Q*1	*Q*2	*Q*3	*Q*4	*Q*5	*Q*6	*Q*7	*Q*8
Q1	1	0.4653	−0.488	−0.5432	−0.0158	−0.1863	0.2503	−0.1696
Q2	0.4653	1	0.0668	0.9769	−0.5286	0.6952	−0.2077	−0.2102
Q3	−0.488	0.0668	1	0.0558	0.1924	0.3128	0.4909	0.1212
Q4	−0.5432	0.9769	0.0558	1	−0.4391	0.6379	−0.262	−0.1649
Q5	−0.0158	−0.5286	0.1924	−0.4391	1	−0.2252	0.5748	0.815
Q6	−0.1863	0.6952	0.3128	0.6379	−0.2252	1	−0.112	0.0534
Q7	0.2503	−0.2077	0.4909	−0.262	0.5748	−0.112	1	0.6568
Q8	−0.1696	−0.2102	0.1212	−0.1649	0.815	0.0534	0.6568	1

**Table 9:** Eigenvalue of the coefficient correlation matrix and their contribution rates.

Principal component	Eigen value	Ratio	Cumulative contribution ratio
1st	3.383	42.2875	42.2875
2nd	2.0843	26.05375	68.34125
3rd	1.547	19.3375	87.67875
4th	0.4842	6.0525	93.73125
5th	0.3082	3.8525	97.73125
6th	0.1309	1.63625	99.22
7th	0.0605	0.75625	99.97625
8th	0.0019	0.02375	100

**Table 10:** Principal component score of coefficient correlation matrix.

QoS attributes	1st	2nd	3rd
Q1	−0.26	0.1475	0.6426
Q2	0.481	−0.2865	0.0331
Q3	−0.1034	−0.3829	0.5942
Q4	0.4725	−0.2914	−0.0405
Q5	−0.4101	−0.3088	−0.2756
Q6	0.3217	−0.4065	0.1751
Q7	−0.3358	−0.4231	0.1033
Q8	−0.2875	−0.4744	−0.3376

**Table 11:** Overall values and ranking of cloud services.

Cloud service	*z*_1_	*z*_2_	*z*_3_	*Z*	Rank
CSP1	−0.0428	−0.0459	0.107	−0.0093	9
CSP2	0.0872	−0.1127	0.1199	0.0307	2
CSP3	0.084	−0.0611	0.1315	0.045	1
CSP4	−0.0915	−0.027	0.134	−0.0198	10
CSP5	0.1724	−0.1632	−0.0118	0.0281	4
CSP6	0.0164	−0.0768	0.0781	0.002	7
CSP7	0.1194	−0.1122	0.0219	0.0255	6
CSP8	−0.0282	−0.0438	0.1077	−0.0025	8
CSP9	0.0872	−0.1127	0.1199	0.0302	3
CSP10	0.0968	−0.16	0.1442	0.0271	5

## Data Availability

The data used to support the findings of this study are available from the corresponding author upon request.
